# Nurses’ Engagement in Digital Health Clinical Trials Using Unobtrusive Monitoring Technologies: Constructivist Grounded Theory Study

**DOI:** 10.2196/90982

**Published:** 2026-09-22

**Authors:** Xiyi Wang, Zhaozhang Sun, Geraldine Lee, Patricia C Dykes, Yun Hu, Wenfang Mu, Chen Zhang, Zhiguo Zou, Li Xu

**Affiliations:** 1 School of Nursing, Shanghai Jiao Tong University, Shanghai, China; 2 Department of Nursing, Renji Hospital, Shanghai Jiao Tong University School of Medicine, No. 160 Pujian Road, Pudong New AreaShanghai, 200127, China, 86 135 0183 0955; 3 Department of Applied Health Sciences, School of Health Sciences, College of Medicine and Health, University of Birmingham, Birmingham, United Kingdom; 4 Centre for National Training and Research Excellence in Understanding Behaviour (CENTRE-UB), Economic and Social Research Council, Department of Applied Health Sciences, School of Health Sciences, College of Medicine and Health, University of Birmingham, Birmingham, England, United Kingdom; 5 Catherine McAuley School of Nursing & Midwifery, Brookfield Health Sciences Complex, University College Cork, Cork, Ireland; 6 Centre for Data Science, Nell Hodgson Woodruff School of Nursing, Emory University, Atlanta, GA, United States; 7 Department of Cardiology, Renji Hospital, Shanghai Jiao Tong University School of Medicine, Pudong New Area, Shanghai, China

**Keywords:** digital health, unobtrusive digital monitoring, engagement, grounded theory, implementation science, clinical trials, health technology adoption

## Abstract

**Background:**

Unobtrusive digital monitoring technologies (UDMTs) enable continuous data collection beyond episodic clinical encounters and are increasingly incorporated into digital health clinical trials. However, their successful use depends on their integration into routine clinical practice. Poor integration can increase hidden nursing workload, disrupt workflows, compromise data quality, and limit the sustainability of digital trials. Although nurses play a central role in coordinating clinical, research, and technological activities, little is known about how they engage with UDMT-enabled clinical trials in everyday practice.

**Objective:**

The study aims to develop a constructivist grounded theory explaining how nurses engage with UDMT-enabled digital health clinical trials within routine clinical practice.

**Methods:**

Using Charmaz’s constructivist grounded theory approach, registered nurses with experience in leading, coordinating, or implementing UDMT-enabled clinical trials were recruited from 2 tertiary teaching hospitals in China through purposive and theoretical sampling. Between February and October 2025, data were generated through semistructured focus groups and individual interviews. Analysis proceeded concurrently with data collection through initial, focused, and theoretical coding, supported by constant comparison, memo writing, and theoretical sampling until theoretical sufficiency was achieved.

**Results:**

Twenty-three nurses from 5 UDMT-enabled clinical trials participated. The analysis generated supported translation theory, conceptualizing nurse engagement as a dynamic and situated process of aligning technological systems, clinical workflows, organizational arrangements, and patient expectations during digital clinical trials. Supported translation is enacted through 4 interrelated and coconstitutive processes: developing readiness for engagement, negotiating organizational conditions, integrating technology into clinical practice, and cultivating socioethical trust. Rather than representing sequential stages, these domains interact dynamically and are sustained through the interpretive, relational, and adaptive work of nurses. The findings demonstrate that implementation is not merely a process of technology adoption but an ongoing accomplishment through which nurses produce the conditions necessary for digital trial delivery.

**Conclusions:**

Supported translation theory offers a practice-based explanation of how nurses engage in UDMT-enabled digital health clinical trials by continuously aligning technological, clinical, organizational, and social elements. The theory extends existing implementation perspectives by specifying the nursing work through which digital health interventions become workable in practice and provides a conceptual foundation for future implementation research, intervention design, and workforce development. Further research should examine and refine the theory across diverse digital health settings and technologies.

## Introduction

Digital health technologies, including mobile health apps, wearable sensors, and real-time monitoring systems, have the potential to transform health care delivery by improving clinical outcomes, enhancing workflow efficiency, and enabling personalized care [1,2]. Unobtrusive digital monitoring technologies (UDMTs), such as smartwatches and sensor-based systems, exemplify this potential by facilitating continuous physiological and behavioral data collection outside traditional clinical encounters [3]. Despite growing evidence supporting their clinical and research value, the integration of UDMTs into routine care and clinical trial workflows remains inconsistent [4,5]. This implementation gap highlights the challenges of embedding digital innovations within complex health care systems, where technological capabilities must be aligned with organizational structures, professional practices, and patient needs.

The increasing number of digital health trials reflects growing confidence in the potential effectiveness of these technologies [6]. However, evidence from implementation science demonstrates that successful integration depends on more than technological performance alone. Factors such as workflow compatibility, organizational readiness, professional norms, technological interoperability, and broader sociocultural contexts influence whether innovations can be effectively incorporated into practice [7-10]. Digital clinical trials should therefore be understood not simply as evaluations of intervention efficacy but as sociotechnical processes through which technologies are enacted within real-world health care environments. Failure to account for these contextual processes may compromise implementation fidelity, data quality, and the translation of research findings into practice [11].

Within these contexts, nurses play a central and operationally critical role in enabling the implementation of digital health technologies [4,12-15]. Their work is not limited to technical facilitation; rather, it includes enrolling and orienting patients to UDMTs, sustaining engagement and adherence, troubleshooting device-related issues, interpreting and contextualizing incoming data, and continuously reconciling protocol requirements with dynamic clinical priorities [16,17]. Through these activities, nurses actively shape how digital technologies become integrated into everyday care. Despite this contribution, nursing work remains insufficiently theorized within digital health research, which has predominantly focused on technological, organizational, or patient-level determinants of implementation [12,18,19]. As a result, limited attention has been paid to how nurses themselves engage with digital technologies and how such engagement influences implementation processes.

Existing studies on digital health adoption have largely been informed by predefined theoretical models, including technology acceptance frameworks, implementation science approaches, and diffusion-oriented perspectives [20,21]. These approaches have generated important insights into the determinants of technology uptake, identifying factors such as perceived usefulness, ease of use, organizational readiness, implementation climate, and behavioral intention [10,22,23]. However, they primarily conceptualize health care professionals as users responding to technological or organizational conditions, with comparatively less attention given to the interpretive, relational, and adaptive work through which digital innovations become integrated into routine care. This limitation is particularly evident in clinical trials involving UDMTs. Nurses do not simply adopt technologies [12]; they continuously interpret data, reconcile protocol requirements with competing clinical demands, adapt workflows, and respond to changing patient circumstances [23,24]. Therefore, engagement appears less as a discrete behavioral outcome than as an ongoing process of interpretive and sociotechnical work. Although complexity, systems, and sociotechnical perspectives have increasingly informed digital health scholarship, empirical theory explaining how nurses accomplish this work remains underdeveloped [25].

Accordingly, there is a need for theory-generating research that explains how frontline nurses construct and sustain engagement with UDMTs within real-world clinical trial environments. Such work can advance the understanding of how nursing practice, professional roles, and professional boundaries are being reshaped as digital technologies become increasingly embedded in clinical research and health care delivery [26,27]. Developing a practice-based explanatory theory is therefore important for clarifying how nurses contribute to the implementation and integration of digitally enabled models of clinical research and care. Constructivist grounded theory (CGT) is particularly well suited to this aim because it supports the development of explanatory theory grounded in participants’ situated practices, interpretations, and interactions rather than the testing of predefined constructs [28,29]. Therefore, this study aimed to develop a CGT explaining how nurses engage with UDMT-enabled digital health clinical trials within routine clinical practice.

## Methods

### Study Design

This qualitative study employed a CGT methodology [29], using focus groups and semistructured interviews to explore nurses’ engagement with UDMTs in clinical trial settings. CGT, as developed by Charmaz, is well suited to investigating complex practice-based phenomena in which meanings and actions are shaped by social and organizational contexts [28]. From a constructivist epistemological position, this study assumes that knowledge is jointly produced through interactions between participants and researchers, rather than discovered as an objective reality. The study is reported in accordance with the COREQ (Consolidated Criteria for Reporting Qualitative Research) and the SRQR (Standards for Reporting Qualitative Research; Checklist 1).

### Study Setting

The study was conducted in 2 tertiary teaching hospitals in China. Both sites were purposefully selected based on their advanced digital health infrastructure and active engagement in clinical trials involving digital health technologies. The 2 hospitals were accredited at Level 6 under the National Health Record Digital Application Maturity framework and designated Level 4 “Smart Hospitals” by the National Health Commission, indicating advanced digital infrastructure and integration of digital health technologies.

Site selection was informed by the research team’s professional networks to facilitate access to diverse UDMT-related trial contexts. The principal investigator (XW) worked with nursing research departments at each institution to identify clinical trial programs that varied in patient populations, intervention characteristics, and implementation contexts, thereby enabling maximum theoretical variation for comparative analysis.

### Participants and Recruitment

Participants were registered nurses employed full-time at the participating hospitals. Inclusion criteria were (1) registered nurse qualifications, (2) direct involvement in clinical trials incorporating UDMTs, and (3) experience in leading, coordinating, or implementing UDMT-related trial activities, including patient-facing and/or device-related responsibilities. Nurses with only indirect or administrative involvement in trials, without direct interaction with patients or UDMT systems, were excluded to ensure relevance to the study aim.

Recruitment was facilitated by 2 investigators (XW and ZZ), who had established collaborations with ongoing digital health research programs. Potential participants were identified through nursing research departments and clinical trial teams and recruited from 5 UDMT-enabled clinical trials across 4 clinical contexts: cardiac critical care, postacute myocardial infarction home monitoring, multidisciplinary electrocardiographic monitoring, and surgical intensive care. Within each context, potential participants were identified through nursing research departments and clinical trial teams. Eligible nurses received an invitation via WeChat from the research team, together with a participant information sheet outlining the study purpose, procedures, confidentiality arrangements, and the voluntary nature of participation. Of the nurses approached, 3 declined participation because of concerns regarding ongoing research commitments or lack of interest. All interviews were conducted in Mandarin Chinese, the participants’ primary working language.

### Sampling

Sampling followed CGT principles [30] and combined initial purposive sampling with subsequent theoretical sampling to support theory development. Initial participants were identified through principal investigators of digital clinical trials at a tertiary hospital in Shanghai. Initial purposive sampling sought information-rich participants with diverse experience implementing UDMT-enabled clinical trials across different clinical settings, enabling early exploration of nurses’ engagement with digital monitoring technologies.

As analysis progressed, theoretical sampling was guided by developing categories rather than participant characteristics alone. Ongoing coding, constant comparison, and memo writing informed decisions about which clinical contexts and participants could best elaborate, challenge, or extend developing interpretations [31]. Rather than increasing sample size within individual settings, sampling deliberately moved across clinical contexts to examine how developing processes operated under different organizational, technological, and care conditions. Recruitment therefore progressed from cardiac critical care (n=6) to postacute myocardial infarction home monitoring (n=10), multidisciplinary electrocardiographic monitoring (n=5), and finally surgical intensive care (n=2), allowing for the refinement of category properties, contextual variation, and theoretical boundaries. Within each context, snowball sampling identified additional nurses able to provide conceptually relevant experiences. In later stages, theoretical sampling focused specifically on disconfirming cases to challenge developing interpretations and strengthen theoretical explanations.

Consistent with CGT, recruitment was guided by conceptual development rather than predetermined sample sizes. Theoretical sufficiency was considered achieved when additional data no longer substantially expanded the properties, relationships, or explanatory dimensions of developing categories, but instead contributed to their refinement and contextual elaboration [32]. Early data analysis indicated that initial categories were sufficiently developed after the first 14 participants, while subsequent interviews were conducted to further examine category variation, relationships, and theoretical relevance across different clinical contexts. With 21 participants, categories demonstrated sufficient conceptual depth and explanatory coherence to support the developing theoretical framework. Two additional nurses from contrasting surgical intensive care unit trial contexts were theoretically sampled to explore disconfirming evidence and clarify the boundary conditions of the theory. A saturation tracking grid documenting code development, category refinement, and progression toward theoretical sufficiency is provided in Table S1 of Multimedia Appendix 1.

### Data Collection Procedures

Between February and October 2025, data were generated through 2 semistructured focus groups and 17 individual semistructured interviews. Interview guides were revised between sampling phases to pursue analytical questions identified through ongoing analysis (Table S2 in Multimedia Appendix 1). All sessions were audio-recorded with participants’ consent.

Focus groups were undertaken during the initial phase to explore shared understandings, collective experiences, and organizational norms related to the implementation of UDMT-related clinical trials. The group format facilitated discussion of common practices, contextual influences, and perceived challenges associated with participation in digital health clinical trials. Insights generated from these discussions informed the development of the initial coding and sensitized the subsequent theoretical sampling. Focus groups were conducted in private meeting rooms within the participating hospitals to ensure confidentiality and minimize workplace interruptions.

Individual interviews were subsequently conducted to deepen conceptual understanding and explore variation in nurses’ experiences across different clinical contexts. Interviews commenced with broad, open-ended questions inviting participants to describe their experiences of implementing and engaging with UDMT-related trial protocols. Practically, preliminary interpretations developed through earlier interviews were introduced during subsequent interviews to invite participants to confirm, challenge, elaborate, or reinterpret developing understandings, thereby supporting the coconstruction of meaning. Interview guides were progressively refined throughout the study to investigate developing concepts such as workflow integration, adaptive practices, interprofessional collaboration, ethical tensions, technological negotiation, patient engagement, and the reconciliation of research requirements with routine clinical care. Overall, interviews were conducted either face-to-face in private locations selected by participants or through secure videoconferencing platforms when in-person meetings were not feasible. Interviews were scheduled outside participants’ clinical responsibilities to minimize disruption to patient care and encourage open discussion.

Field notes were documented during and immediately following each focus group and interview to capture contextual observations, nonverbal interactions, developing analytic insights, and reflections on researcher-participant interactions. Audio recordings were transcribed verbatim in Mandarin Chinese using AI-assisted transcription software (Tencent Meeting, Tencent Technology) and verified against the original recordings by the first author (XW). Data analysis was conducted in Mandarin Chinese to preserve linguistic, contextual, and conceptual nuance.

### Researcher Characteristics and Reflexivity

All focus groups and interviews were conducted by the first author (XW), a female PhD-prepared nurse researcher with expertise in digital health, clinical trials, and qualitative research. She had no supervisory or managerial relationship with participants. Interpretive rigor was further enhanced through investigator triangulation. A female senior cardiac nurse specialist (LX) and a male PhD-trained cardiologist (ZZ) independently reviewed selected transcripts, coding decisions, analytic memos, and category development. Consistent with a CGT approach, researcher subjectivity was viewed as an analytic resource requiring ongoing critical reflection rather than elimination. The research team’s professional backgrounds in nursing and clinical research informed both data generation and interpretation and were critically examined throughout the study through reflexive journaling, field notes, theoretical memo writing, and regular analytic discussions [33,34].

Divergent interpretations were treated as opportunities for theoretical inquiry rather than discrepancies requiring consensus, enabling deeper conceptual exploration and refinement. The developing conceptual interpretations were also discussed with selected participants to explore their resonance with participants’ experiences and to further refine the developing theoretical explanation. Rigor was further enhanced through theoretical sampling, constant comparative analysis, maintenance of an audit trail, reflexive memo writing, investigator triangulation, participant reflection, and the inclusion of theoretically selected disconfirming cases [35].

### Data Analysis

Analysis commenced after the first focus group and proceeded iteratively alongside data generation through initial, focused, and theoretical coding [36]. All data sources were managed using Microsoft Word and Excel to support systematic coding and conceptual development. To preserve confidentiality while maintaining analytic traceability, all participants were assigned anonymized identifiers. Interview participants were referred to by case and participant number (eg, C2-RN1).

Initial coding was conducted line-by-line and remained close to participants’ language, actions, and experiences. Particular attention was given to participants’ practice-oriented descriptions of trial work, which frequently emphasized clinical responsibilities, workflow demands, patient care priorities, and professional accountability rather than formal informatics terminology. Constant comparative analysis was undertaken throughout this stage to examine similarities and differences across incidents, participants, and clinical contexts [35]. To enhance reflexive interpretation, 2 researchers (XW and LX) independently coded a subset of transcripts before discussing their interpretations. Differences in coding were treated as sites of analytic inquiry rather than discrepancies requiring resolution. The researchers’ complementary disciplinary perspectives, one oriented toward theoretical abstraction and the other toward clinical practice, supported the critical interrogation of assumptions and enhanced theoretical sensitivity.

Focused coding involved selecting the most analytically significant initial codes and synthesizing them into higher-level conceptual categories. Analytic memos documented developing interpretations, explored relationships among categories, and generated questions that guided subsequent theoretical sampling. Category properties and dimensions were progressively refined through constant comparison across clinical settings, organizational arrangements, and technological contexts. Cases from contrasting implementation contexts, including elaborative cases from postacute myocardial infarction home-monitoring programs and theoretically selected disconfirming cases from surgical intensive care unit clinical trials, were examined to refine category boundaries, clarify conditional variation, and strengthen the explanatory power of the developing theory (see Multimedia Appendix 2).

Theoretical coding was subsequently undertaken to integrate categories into a coherent explanatory framework. Consistent with the Charmaz constructivist approach, theoretical coding remained an interpretive process rather than a formulaic procedure. The NURSE (navigating uncertainty, mobilizing resources, seeking support, and sustaining empowerment) heuristic was developed during the final stages of the analysis after core conceptual categories had been established (Multimedia Appendix 3). Derived inductively from recurring cross-case patterns, it served as a heuristic for synthesizing and communicating the substantive theory rather than directing coding or category development.

As theoretical categories became increasingly developed, concepts from implementation science and complexity-informed scholarship, including normalization process theory, sociotechnical systems theory, and complex adaptive systems perspectives, were consulted to enhance theoretical sensitivity and support conceptual refinement (Multimedia Appendix 4). These perspectives were neither imposed on the data nor used as deductive frameworks; rather, they supported conceptual refinement and enhanced analytic sensitivity during later stages of theory construction.

The final outcome of the analysis was a substantive grounded theory explaining how nurses engage in processes of “supported translation” within UDMT-enabled clinical trials. Selected quotations were translated into English and independently reviewed by bilingual members of the research team (XW, ZZ, WM, and ZS) to ensure semantic and conceptual fidelity to participants’ meanings within the limits of translation.

### Ethical Considerations

Ethical approval was obtained from the ethics committees of Renji Hospital, Shanghai Jiao Tong University School of Medicine (approval number LY2023-175-B) and the Second Affiliated Hospital of Zhejiang University School of Medicine (approval number 20241288). The study was conducted in accordance with institutional regulations and the principles of the Declaration of Helsinki. To compensate participants for their time, each received RMB 100 per hour (approximately US $15). All participants were provided with written and verbal information about the study and gave written informed consent prior to participation. Participation was voluntary, and participants could withdraw at any time without consequence. Data were stored securely on password-protected devices accessible only to members of the research team.

## Results

### Participant Characteristics

Twenty-three registered nurses participating in 5 clinical trials involving UDMTs were interviewed (Table 1). Interview durations ranged from approximately 30 to 65 minutes, while focus groups lasted approximately 57 minutes each.

**Table 1.** Participants’ characteristics (N=23)^a^.

Participants^b^	Job professionals	Education level	Years of work experience	Role and functions
C1-RN1	Nurse manager	Master	16	Leading role; liaison with multidisciplinary team
C1-RN2	Clinical nurse	Three-year diploma	28	Frontline implementer; direct patient communication
C1-RN3	Clinical nurse	Bachelor	8	Frontline implementer; direct patient communication
C1-RN4	Clinical nurse	Three-year diploma	14	Frontline implementer; direct patient communication
C1-RN5	Clinical nurse	Bachelor	6	Frontline implementer; direct patient communication
C1-RN6	Clinical nurse	Bachelor	11	Frontline implementer; direct patient communication
C2-RN1	Clinical nurse educator	Bachelor	25	Coordinator and facilitator role; liaison with multidisciplinary team
C2-RN2	Clinical research nurse	Master	2	Coordinator and facilitator role; liaison with multidisciplinary team
C2-RN3	Clinical nurse	Bachelor	18	Frontline implementer; direct patient communication
C2-RN4	Clinical nurse	Bachelor	8	Frontline implementer; direct patient communication
C2-RN5	Clinical nurse	Three-year diploma	4	Frontline implementer; direct patient communication
C2-RN6	Clinical nurse	Three-year diploma	4	Frontline implementer; direct patient communication
C2-RN7	Clinical nurse	Bachelor	3	Frontline implementer; direct patient communication
C2-RN8	Clinical nurse	Bachelor	9	Frontline implementer; direct patient communication
C2-RN9	Clinical nurse	Bachelor	9	Frontline implementer; direct patient communication
C2-RN10	Clinical nurse	Three-year diploma	15	Frontline implementer; direct patient communication
C3-RN1	Nurse manager	Bachelor	17	Leading role; liaison with multidisciplinary team
C3-RN2	Clinical research nurse	Bachelor	6	Frontline implementer; direct patient communication
C3-RN3	Clinical research nurse	Bachelor	4	Frontline implementer; direct patient communication
C3-RN4	Clinical research nurse	Bachelor	4	Frontline implementer; direct patient communication
C3-RN5	Clinical research nurse	Bachelor	3	Frontline implementer; direct patient communication
C4-RN1	Clinical nurse	Bachelor	6	Coordinator and facilitator role
C5-RN1	Clinical nurse	Bachelor	8	Frontline implementer; direct patient communication

a There were 18 (78.3%) frontline implementers, 3 (13.0%) coordinators/facilitators, and 2 (8.7%) nurse leaders.

b Participant identifiers are anonymized to preserve confidentiality. C1-C5 denote the 5 digital clinical trial contexts (cases), and RN1, RN2, etc, indicate individual registered nurse participants within each case. For example, C1-RN1 refers to the first nurse participant recruited from digital clinical trial case 1. Regarding the education level, in the Chinese nursing education system, a bachelor's degree refers to a 4-year university nursing program, whereas a 3-year nursing diploma refers to a 3-year college-level nursing program. Clinical experience is reported as years of nursing practice.

Participants were predominantly female (n=22, 95.7%). Their clinical experience ranged from 2 to 28 years, with a mean of 9.9 (SD 7.1) years and a median of 8 (IQR 4-15) years. Most participants held a bachelor’s degree (n=16, 69.6%), followed by a 3-year nursing diploma (n=5, 21.7%) and a master’s degree (n=2, 8.7%). Most participants served as frontline implementers (n=18, 78.3%), assuming primary responsibility for patient communication, device management, and operational reporting. The remaining participants held coordinator and facilitator roles (n=3, 13.0%), supporting protocol adherence and training, or leadership roles (n=2, 8.7%), overseeing team coordination and trial objectives.

### Constructing Supported Translation Theory

#### Overview of the Core Category

**Figure 1.**
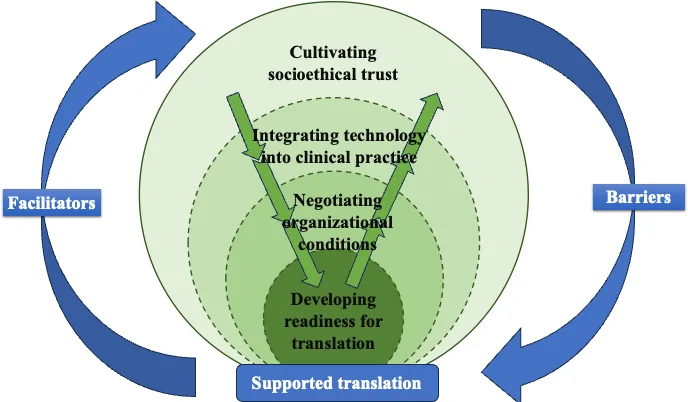
The overview of supported translation theory.

The analysis generated a substantive grounded theory of supported translation, explaining how nurses actively produce workable alignment between UDMT-enabled clinical trial protocols and the contingencies of everyday clinical practice (Figure 1).

Supported translation is a nonlinear, recursive, and socially situated process through which externally defined digital trial protocols are rendered locally actionable within dynamic clinical environments. Rather than implementing protocols, nurses actively translate them through ongoing interpretive, relational, and material work that responds to persistent tensions between standardization demands and clinical variability. At the core of this process is a conceptual patterning referred to as the NURSE heuristic, which captures recurring modes of action: navigating uncertainty, mobilizing resources, seeking support, and sustaining empowerment. The NURSE heuristic is not a stage model but an analytically constructed sensitizing device that expresses patterned yet situationally variable practices through which translation work is accomplished.

At its core, supported translation is the situated production of feasibility. Feasibility is not assumed or given; it is actively accomplished through iterative cycles in which nurses interpret ambiguous requirements, negotiate constraints, and stabilize workable routines within shifting clinical conditions. Through this process, digital trials become temporarily and contingently “doable” in practice. Supported translation is further grounded in four interrelated analytic dimensions: (1) developing readiness for translation, (2) negotiating organizational conditions, (3) integrating technology into clinical practice, and (4) cultivating socioethical trust. These domains are not sequential stages but intersecting arenas of action that continuously coproduce the conditions under which translation work becomes possible (Table 2).

**Table 2.** Supported translation theory (categories, properties, and processes).

Category	Properties	Processual meaning
Developing readiness for translation	Appraising clinical value, developing interpretive competence, engaging in experiential learning, and recalibrating professional identity	Producing individual and professional preparedness for engaging with digital trial work
Negotiating organizational conditions	Legitimizing leadership, structuring resources, integrating workflow, and coordinating interprofessional teams	Constructing institutional conditions that enable or constrain translation work
Integrating technology into clinical practice	Negotiating usability, adapting contextually, evaluating clinical utility, and mediating feedback	Enacting situated embedding of UDMTs^a^ within routine care practices
Cultivating socioethical trust	Building relational trust, enacting transparency work, engaging in ethical sense-making, and responding to equity divides	Establishing moral and relational legitimacy for sustained participation
Supported translation (core category)	Navigating uncertainty, mobilizing resources, seeking support, and sustaining empowerment.	Producing workable coherence between digital trials and clinical reality

a UDMTs: unobtrusive digital monitoring technologies.

#### Category: Developing Readiness for Translation

Developing readiness for translation captures the ongoing constitution of cognitive, affective, and professional capacities required to engage with UDMT-enabled clinical trials. In contrast to a preexisting competency, readiness was constructed as a processual outcome generated through repeated exposure to technological, organizational, and clinical demands.

A foundational element of readiness was clinical value appraisal, through which nurses justified participation based on perceived patient benefit and relevance to safety:

*This was why I accepted the trial…monitoring devices are important and significant for these patients.*

[C1-RN2]

Readiness was further enacted through the development of interpretive competence, enabling nurses to translate technical protocols into patient-accessible communication while maintaining procedural fidelity. This involved anticipatory judgment regarding communicative risk and context-sensitive adaptation:

*I am afraid that if I said something wrong it could compromise the project. Therefore, my colleagues and I practice how to present the project instructions, because each patient is different.*

[C1-RN4]

Through iterative practice, nurses gradually shifted from uncertainty toward embodied confidence, developing what can be conceptualized as experiential understanding of device functionality, patient responses, and procedural variability. This experiential learning also contributed to professional identity recalibration, as nurses repositioned themselves as hybrid clinical actors engaged in coordination, education, and informal research mediation.

However, readiness remained structurally uneven. Some participants articulated persistent gaps in methodological participation and research capability:

*We are not yet fully prepared for nursing research… our role was largely reactive, and our research skills and outputs remained insufficient.*

[C3-RN3]

Therefore, developing readiness represented an ongoing process of learning, sense-making, and identity reconstruction that enabled nurses to reduce uncertainty and translate digital research protocols into patient-centered clinical action.

#### Category: Negotiating Organizational Conditions

Negotiating organizational conditions refers to the institutional work required to secure, stabilize, and sustain the conditions for translation. Rather than acting as a passive backdrop, organizational structures actively shaped what translation work could be accomplished.

A key enabling mechanism was leadership legitimization, whereby visible endorsement from senior nurses or managers normalized engagement and reduced ambiguity surrounding trial participation:

*During the implementation of this scheme, it was a process of continuous experience accumulation…we had to prepare for a wide range of details*.

[C3-RN1]

Organizational support also materialized through training opportunities, resource allocation, and workflow integration. When responsibilities of UDMTs were embedded into existing routines, translation work became more sustainable. In contrast, when additional responsibilities were layered onto care without structural adjustment, nurses experienced role strain and workload inflation:

*Every shift I must charge it, keep it safe…the workload has definitely increased*.

[C2-RN6]

A further dimension involved interprofessional role negotiation, particularly where physicians’ variable engagement redistributed explanatory and coordination burdens onto nurses. This produced asymmetries in translational labor:

*The personality of cardiovascular patients is often very stubborn; more explanation work of the trial would be better handled by the physician*.

[C1-RN3]

At its most destabilizing extreme, organizational discontinuity, particularly the withdrawal of funding, resulted in the collapse of translation work altogether:

*The project suddenly stopped because the related funding application was unsuccessful…. The devices are now stored in a cabinet and are no longer being used*.

[C5-RN1]

Thus, organizational conditions operate not as background context but as active structuring forces that enable, redistribute, or terminate translation practices.

#### Category: Integrating Technology Into Clinical Practice

Integrating technology into clinical practice captures the situated enactment of UDMTs through ongoing adaptation and interpretive mediation within workflow constraints. Integration was contingent on perceived clinical utility and usability, which shaped whether devices were meaningfully embedded or superficially adopted.

Where technologies produced clinically actionable insights, integration was more robust:

*The smartwatch definitely complements the weakness of ECG devices in the CCU, especially for sleep monitoring. It provides exact data and evidence to guide patient education, rather than relying only on patients’ self-reports, which are sometimes inaccurate.*

[C2-RN2]

However, integration frequently remained partial when feedback loops between data collection and clinical decision-making were weak, resulting in procedural rather than clinical engagement:

*We usually just put the smartwatch on the patient… feedback to patients was actually missing.*

[C5-RN1]

In response, nurses engaged in technological mediation work, including troubleshooting, reeducation, and translation of technical outputs into actionable clinical meaning. This work extended beyond device handling to the coordination of patient-family-technology relations.

*But once they returned home, myriad problems emerged… We then have to persuade their children to visit them and help reconnect the network… [If they are busy], we have to make video calls to teach the patients… This is the kind of repetitive, challenging process we constantly deal with.*

[C3-RN1]

Accordingly, integration was not adoption but the ongoing coproduction of technological functionality within clinical systems, sustained through continuous human adjustment.

#### Category: Cultivating Socioethical Trust

Cultivating socioethical trust describes the relational and moral work through which the legitimacy of participation in UDMT-enabled trials is produced and maintained. This category moves beyond technical or clinical compliance, framing trust as a dynamic “relational infrastructure” that aligns patients, clinicians, and institutions through moral and communicative consensus.

A primary mechanism was transparent sense-making, whereby nurses translated complex technological systems into ethically intelligible narratives that reduced perceived surveillance risks and addressed uneven digital literacy. This was particularly salient among older patients, for whom technological monitoring required careful framing as care rather than control. Trust was also maintained through relational endurance work, particularly in managing patient fatigue and withdrawal tendencies over time. When the monotony of daily tracking eroded patient motivation, nurses deployed empathetic and persistent relational care to sustain cooperation:

*Furthermore, high patience is mandatory because our patients are generally older… you must communicate in a way they can easily understand… Some patients eventually grew weary of the routine and became reluctant. But if you communicated gently, they would agree to continue*.

[C3-RN5]

Trust was frequently grounded in preexisting therapeutic relationships, with trust in clinicians functioning as a prerequisite for acceptance of technological monitoring. Existing therapeutic relationships enabled nurses to frame digital monitoring as a supportive extension of care rather than an intrusive intervention:

*I think the biggest motivator is really the patient’s needs…one patient came up to me and said, “I want a smartwatch that lets the doctor see my data, so we can talk directly about my illness.”*

[C2-RN1]

Importantly, trust was contingent on system responsiveness. Timely technical support reinforced institutional credibility, whereas delays risked destabilizing participation:

*[When] we could respond efficiently and help them troubleshoot, I believe their compliance increased-not in terms of blind obedience, their trust and confidence in us became stronger.*

[C3-RN4]

Thus, socioethical trust was observed as a continuously enacted relational accomplishment, critical for stabilizing participation in technologically mediated clinical trials.

## Discussion

### Principal Findings

This study developed supported translation theory, a processual explanation of how nurses make UDMT-enabled clinical trials workable within routine clinical practice. Rather than conceptualizing implementation as the adoption of predefined interventions [19], the findings suggest that implementation is an ongoing process of translation through which nurses continuously align technological systems, research protocols, organizational arrangements, and patient care needs.

Supported translation reframes nurses as active agents who mediate coherence across multiple and often competing demands. Through interpretive, relational, and material work, nurses transform externally defined trial requirements into locally feasible clinical practices. Feasibility is therefore not an inherent property of UDMT interventions but a temporary and situated accomplishment produced through interactions among technologies, health care professionals, patients, and organizational systems. By explicating the work through which digital interventions become operational in practice, this theory extends implementation and complex systems’ perspectives beyond adoption toward the processes that sustain implementation over time [37].

Readiness is commonly conceptualized as a condition present before digital health implementation that influences early implementation outcomes [38]. Our findings extend this view by showing that practical readiness also developed during implementation. Nurses initially described uncertainty about device use, protocol requirements, and changing clinical responsibilities. Through repeated engagement with patients, technologies, and colleagues, they learned to interpret device-generated information, adapt workflows, negotiate responsibilities, and mobilize technical and organizational support [10,39,40]. Importantly, this learning was accompanied by an expansion of their professional roles. Nurses increasingly assumed hybrid roles involving patient education, coordination, and research facilitation, consistent with recent descriptions of nurses as digital intermediaries or navigators within technology-mediated care environments [4,39]. However, opportunities for methodological participation and knowledge production remained uneven. Therefore, supported translation conceptualizes practical readiness as an ongoing, relational process shaped by experience, professional roles, and access to organizational and technical support, rather than solely as a fixed individual attribute assessed before implementation.

Organizational conditions were similarly identified as critical determinants of implementation. Beyond functioning as contextual background, organizational structures actively legitimized, constrained, and redistributed the work required to sustain digital trials [41]. Leadership support and managerial endorsement facilitated engagement, whereas limited preparation for digital transformation often required nurse leaders to develop adaptive strategies through experience [42]. The sustainability of implementation further depended on the extent to which trial activities were integrated into existing workflows. When digital responsibilities were embedded within routine practice, participation became more manageable. Conversely, when additional responsibilities were introduced without corresponding organizational support, nurses experienced increased workload, invisible labor, and emotional burden, consistent with previous implementation research [43]. Moreover, funding discontinuity and infrastructural instability could rapidly destabilize previously established practices, indicating that long-term implementation depends not only on technological effectiveness but also on sustained organizational investment [44]. Future implementation research would therefore focus on identifying leadership and organizational strategies that support translational work while reducing hidden workload.

The findings also demonstrate that technological integration is a coproduced process shaped by interactions between system design, clinical practice, and organizational context, reflecting implementation science principles that balance fidelity with pragmatic adaptation [45-47]. However, technological integration often remained incomplete when data collection was disconnected from clinical decision-making. To compensate for these limitations, nurses undertook substantial mediation work, including troubleshooting, patient education, and coordination with family members. Similar forms of sociotechnical work have been described in previous digital health studies [13,19]. These findings suggest that technological functionality is not solely determined by design characteristics but is continuously produced through situated interactions among technologies, users, and clinical environments. Nurses therefore function not simply as end users but as active contributors to technological performance and clinical value [4,12]. Future studies may usefully examine how co-design approaches and sustained nurse involvement influence long-term usability, workflow integration, and implementation outcomes.

Another important theoretical contribution is the identification of socioethical trust as a relational infrastructure underpinning sustained participation. Trust was not observed as a stable individual attribute but as an ongoing accomplishment produced through transparent communication, ethical reassurance, and therapeutic relationships. Patients’ willingness to engage depended on adequate explanations, responsiveness, and confidence in both health care professionals and health care organizations [5,48,49]. Although increasing familiarity with digital technologies within the Chinese health care context may facilitate the acceptance of mHealth interventions, technological familiarity alone was insufficient to sustain participation [50,51]. Concerns regarding digital inequality and unequal access highlighted the ethical dimensions of implementation. These findings suggest that trust, equity, and inclusion are integral components of digital implementation rather than peripheral considerations [50,52]. Further research could therefore explore how socioethical trust operates across different cultural, organizational, and technological contexts.

### Innovation, Contributions, and Real-World Implications

Supported translation advances the understanding of digital clinical trial implementation by conceptualizing nurses’ engagement as situated alignment work through which UDMT-enabled interventions become clinically, organizationally, and socially workable. Existing implementation research has largely focused on determinants associated with adoption and uptake [7-10]. For example, technology acceptance research has highlighted perceived usefulness, ease of use, and behavioral intention as key influences on technology adoption. Organizational readiness research has emphasized collective commitment and implementation capability as prerequisites for change. Normalization process theory and sociotechnical perspectives have further demonstrated that successful integration of innovations depends on collective work and interactions among people, technologies, and organizational arrangements. Building upon these perspectives, rather than replacing them, supported translation specifies the nursing practices through which UDMT-enabled trial activities are rendered workable, including interpreting device-generated information, adapting clinical routines, negotiating responsibilities, and coordinating support across clinical, technical, and organizational boundaries [39,53]. By elucidating the translational mechanisms through which digital innovations are interpreted, adapted, integrated, and sustained within real-world care environments, supported translation provides a theoretical explanation of how implementation is shaped through dynamic interactions among technologies, clinical practices, organizational structures, and professional roles. This contribution extends implementation research beyond determining whether innovations are adopted toward understanding how they are made workable and sustained within complex health care systems.

Supported translation also advances nursing science by reconceptualizing nurses as active translational agents in digital health implementation. Unlike conventional clinical trials, in which nursing roles are often centered on protocol adherence, participant recruitment, and outcome assessment, digitally enabled trials expand research nursing responsibilities and require continuous coordination among technologies, data flows, clinical decision-making, and patient engagement [15,54]. Nurses contribute not only to intervention delivery and participant support but also to interpreting digital information, resolving technology-related challenges, adapting workflows, and maintaining patient trust in digital systems [23,40]. By positioning nurses as active contributors who shape implementation processes rather than as passive technology users or research personnel, supported translation highlights the importance of nursing expertise and leadership across digital intervention development, clinical trial design, implementation planning, and evaluation. Specifically, the theory explains how nurses connect heterogeneous elements by translating device outputs into clinical meaning, clinical priorities into technical requirements, and protocol expectations into feasible everyday practices. Thus, the central contribution of this study to digital health lies in demonstrating how nurses mediate relationships among data, devices, participants, workflows, and professional groups after digital technologies are introduced into clinical environments.

These findings have important implications for practice and implementation planning. Nurses should be meaningfully involved in decisions regarding device selection, protocol development, workload assessment, staffing models, training strategies, escalation pathways, and longer-term implementation infrastructure. As a practice-based theory, supported translation recognizes that translation processes are shaped by contextual conditions, including organizational capacity, leadership structures, technological maturity, and sociocultural expectations, rather than assuming uniform pathways of implementation. Future research can extend this framework by examining how alignment processes operate across diverse health care systems, clinical contexts, and digital technologies using comparative, longitudinal, and interventional study designs. In practice, involving nurses from the earliest stages of intervention development and implementation planning may enhance clinical feasibility, workflow compatibility, and patient acceptability. Furthermore, theory-informed education and workforce development initiatives may strengthen nurses’ roles as digital health navigators, implementation partners, and research collaborators, thereby enhancing health care systems’ capacity to achieve equitable and sustainable digital transformation.

### Limitations

Several limitations should be considered when interpreting these findings. First, the research was conducted in hospital settings characterized by relatively advanced digital infrastructure. This may constrain the transferability of supported translation theory to contexts with lower levels of technological maturity. Although rigorous qualitative methods were employed to refine abstract theoretical constructs and capture how nurses actively construct workable conditions for participation in digital clinical trials, further research is needed to examine how these processes manifest in resource-constrained or less digitally integrated health care environments.

Second, the sample primarily comprised nurses working in cardiovascular clinical trial units and intensive care settings. The absence of perspectives from physicians, patients, and technology developers limits the extent to which broader sociotechnical and multidisciplinary dynamics can be fully accounted for. Given that nurses often function as key boundary actors across clinical, organizational, and technological domains, future digital health implementation research would benefit from multistakeholder designs that capture the distributed and collaborative nature of implementation work.

Third, consistent with the aims of CGT, this study prioritized the development of a contextually grounded explanatory framework rather than hypothesis testing or variable-level inference. Consequently, the relationships articulated within supported translation theory should be understood as interpretive and conceptual rather than statistically validated. Future research should extend and empirically test the theory using mixed methods, quantitative, and intervention-based designs to assess its applicability, robustness, and explanatory power across diverse health care settings.

### Conclusion

This study develops supported translation theory, a CGT that explains how nurses make UDMT-enabled clinical trials workable in everyday practice. The theory conceptualizes implementation not as a discrete act of technology adoption but as an ongoing process of situated translation through which nurses align technological systems, research protocols, clinical workflows, and patient needs. Supported translation contributes to nursing science by extending the understanding of nurses’ roles in digital health implementation beyond protocol delivery and technology use, highlighting their involvement in the translation and adaptation of digital innovations within practice contexts. As a substantive practice-based theory, it provides a processual account of how digital clinical trials are materially, relationally, and ethically accomplished, offering a theoretical foundation for future research and a practical lens for guiding digitally enabled health care implementation. Future research should further refine and examine the theory across diverse digital health settings to establish its explanatory utility and practical relevance.

## Supplementary material

10.2196/90982 Multimedia Appendix 1 Theoretical sampling trajectory and category development and interview outlines.

10.2196/90982 Multimedia Appendix 2 Reflective memos and case analysis.

10.2196/90982 Multimedia Appendix 3 The NURSE (navigating uncertainty, mobilizing resources, seeking support, and sustaining empowerment) heuristic framework.

10.2196/90982 Multimedia Appendix 4 Application of sensitizing frameworks in theoretical integration and refinement.

10.2196/90982 Checklist 1 COREQ and SRQR checklists.

## Abbreviations

CGT: constructivist grounded theory
COREQ: Consolidated Criteria for Reporting Qualitative Research
NURSE: navigating uncertainty, mobilizing resources, seeking support, and sustaining empowerment
SRQR: Standards for Reporting Qualitative Research
UDMT: unobtrusive digital monitoring technology
