# Supplementary material for: Nurses’ Engagement in Digital Health Clinical Trials Using Unobtrusive Monitoring Technologies: Constructivist Grounded Theory Study
Source: J Med Internet Res. 2026 Sep 22;28:e90982. doi: 10.2196/90982 (PMC13596759; doi:10.2196/90982)
Supplement: Supplementary file 1 [file jmir-v28-e90982-s001.docx]

**Multimedia Appendix 1**

**Table S1: Theoretical sampling trajectory and category development**

| **Analytical phase** | **Emerging analytical gap identified through constant comparison** | **Theoretical sampling decision** | **Theoretical contribution to category development** | **Data source** |
| --- | --- | --- | --- | --- |
| Initial category construction | Limited understanding of how nurses experience and make sense of UDMT-supported care, and no conceptual categories existed to explain technology-mediated nursing practice. | Sample nurses with direct experience using UDMTs in routine cardiac care to generate initial categories and sensitizing concepts. | Generated preliminary categories concerning technology-mediated awareness, workflow adaptation, device reliance, and perceived benefits and burdens. | Two focus groups (n=6), critical cardiac units, Shanghai |
| Category elaboration and process development | Emerging categories described experiences but did not adequately explain how nurses interpret, evaluate, and act upon device-generated information in practice. | Sample nurses working in home-based monitoring contexts where interpretation and response to UDMT data occurred outside immediate clinical supervision. | Refined processual categories explaining how device signals are interpreted, negotiated, and translated into clinical action. Identified mechanisms linking technological information to nursing decision-making. | Semi-structured interviews (n=10), home-based AMI follow-up, Shanghai |
| Theoretical refinement through variation sampling | Relationships between technology use and nursing action were evident, but the conditions shaping these relationships remained insufficiently developed. Organizational and interprofessional influences were not yet theoretically explained. | Purposefully sample multidisciplinary monitoring teams to explore variation in professional roles, communication structures, and organizational arrangements. | Elaborated category properties and dimensions related to interprofessional collaboration, organizational support, role negotiation, and sociotechnical integration. Clarified contextual conditions influencing category relationships. | Additional interviews (n=5), multidisciplinary ECG monitoring teams, Hangzhou |
| Boundary testing and theoretical integration | Core categories were well developed, but their explanatory range and boundary conditions remained uncertain. It was unclear whether identified mechanisms operated similarly in more acute and technologically intensive settings. | Negative cases: Sample nurses working in SICU monitoring systems and early-warning environments to test theoretical robustness under contrasting conditions. | Refined theoretical relationships, specified scope conditions, and confirmed the explanatory limits of the emerging theory. No new category properties or relationships were identified. | Final interviews (n=2), SICU sleep monitoring and early-warning systems |

**Note.**
Theoretical sampling was conducted iteratively and was guided by gaps identified during coding, memo-writing, and constant comparative analysis. Sampling decisions were made by three researchers (XW, XL, and ZZ) to elaborate underdeveloped category properties, explore variation in category relationships, and test emerging theoretical explanations. Clinical settings were selected because they enabled examination of specific analytical questions rather than to achieve contextual representation. Sampling ceased when additional data no longer contributed new theoretical properties, dimensions, or relationships, indicating theoretical sufficiency.

**Table S2. Interview outlines**

| Qualitative interview | Interview outlines | Notes for next round |
| --- | --- | --- |
| Two focus groups (n=6), critical cardiac units, Shanghai | Can you tell me about your first experiences of participating in this project?  What did you think this project was trying to achieve?  How did you feel when the project was first introduced?  In what ways, if any, did the project affect your daily work?  What aspects of the project were easy or difficult to manage?  How did you and your colleagues respond to these challenges?  Have your views about the project changed over time? If so, how?  Can you recall any experiences that influenced your thinking?  Did the project affect how you cared for patients?  How did the wearable devices fit into your usual routines?  What adjustments were needed to make the project work?  How do you see the future of this kind of technology in your practice?  What would need to happen for this work to continue?  How did the project affect staffing and workflow arrangements?  What kinds of support were needed to make the project work?  How were responsibilities negotiated within the team? | Q: How do nurses move from perceiving wearable-device implementation as an additional research burden to viewing it as a meaningful and clinically valuable component of nursing practice? Purpose: Initial category building (open coding and axial coding)  Identify early-stage constructs: burden perception, adaptive efforts, value emergence, and workflow negotiation  Support core category development: value reconstruction under workload pressure; progressing engagement |
| Semi-structured interviews (n=10), home-based AMI follow-up, Shanghai | 1. Value reconstruction trajectory  • How has your understanding of the wearable project changed over time?  • What events or interactions influenced this change?  • What aspects remain problematic or unresolved?  2. Trust, safety, and relational continuity  • How did continuous monitoring influence your sense of safety and reassurance?  • What role did nurse-patient communication play in shaping trust?  • What factors led to trust or distrust in the system?  3. Professional recognition & identity  • How should nursing contributions in digital/monitoring projects be recognized?  • What forms of recognition (clinical, academic, institutional) are meaningful?  • How does recognition influence engagement and sustained participation?  4. Role evolution & competency shift  • How has wearable monitoring changed nursing roles in follow-up care?  • Do nurses function as data interpreters or care coordinators? Why?  • What new competencies are required (technical, interpretive, relational)?  5. Workload redistribution & unintended consequences  • What new responsibilities or hidden workloads emerged?  • How were these managed within existing care structures?  Other explorational questions:  What conditions support long-term integration of this type of care?  How should responsibilities be distributed among professionals?  How should success be evaluated? | Q: How is wearable-generated data transformed into sustainable nursing value across hospital, patients, and digital care ecosystems?  Purpose: Theory refinement and saturation testing, to refine and elaborate the emerging theory of how wearable technologies become integrated into nursing practice and digital healthcare systems.  Strengthen higher-order categories: trust formation, identity reconstruction, institutional embedding  Advance core process: from episodic use to relational continuity and institutionalized value |
| Additional interviews (n=5), multidisciplinary ECG monitoring teams, Hangzhou | 1. Adoption motivation & continuation dynamics  • What motivated initial adoption of wearable monitoring systems?  • What factors influenced continued use or discontinuation?  • How did perception evolve with increased exposure to data?  2. Role redefinition & data mediation  • How has wearable monitoring changed your professional role?  • Do you see yourself as a clinical decision-maker, data interpreter, or system intermediary?  • How is clinical authority negotiated around wearable-generated data?  3. Digital capability & workflow integration  • What digital competencies are essential for effective use?  • How is data interpreted and validated in multidisciplinary teams?  • What barriers exist to cross-system data sharing?  4. Organizational governance & responsibility allocation  • Who should be responsible for interpreting and acting on wearable data?  • What governance model ensures safe and efficient use?  5. Sustainability & institutionalization  • What reimbursement or financing models are feasible?  • What evidence is required for scaling up implementation?  • How should liability and risk be managed across stakeholders? | Purpose: Theoretical sampling for boundary expansion  Test cross-context transferability (hospital, patients in the home, and multidisciplinary systems)  Refine category: “data-mediated role reconfiguration in digital care ecosystems” |
| Final interviews (n=2), SICU sleep monitoring and early-warning systems | • Under what conditions does wearable monitoring fail to produce perceived value?  • What situations lead to resistance, disengagement, or rejection?  • When does continuous monitoring create overload or alert fatigue?  • What breakdowns occurred in communication or workflow integration?  • How are alerts interpreted and acted upon in real time?  • What limits clinical trust in algorithmic outputs?  • What barriers made implementation difficult?  • Were there any unintended consequences for patients or staff? | Q: Under what conditions does engagement become difficult, fragile, or unsustainable?  Purpose:   1. Negative case analysis & theoretical boundary testing 2. Delimit theory scope: when engagement and value reconstruction does NOT occur. 3. Strengthen rigor via deviant case comparison |

Note: In keeping with Charmaz's constructivist grounded theory methodology, interview guides evolved iteratively throughout the study in response to emerging analyses and ongoing interactions between researchers and participants. Questions served as flexible prompts rather than a fixed protocol, allowing participants' experiences and meanings to shape subsequent data generation and theoretical sampling decisions.
