# Supplementary material for: Nurses’ Engagement in Digital Health Clinical Trials Using Unobtrusive Monitoring Technologies: Constructivist Grounded Theory Study
Source: J Med Internet Res. 2026 Sep 22;28:e90982. doi: 10.2196/90982 (PMC13596759; doi:10.2196/90982)
Supplement: Supplementary file 2 [file jmir-v28-e90982-s002.docx]

**Appendix 2. Reflective Memos and Negative Case Analysis (translated from Chinese)**

**A3.1 Reflective Memos (Illustrative Elaborative Cases)**

**Overview:** An important adaptive case emerged from interviews describing the implementation of a wearable home care monitoring project in a critical cardiac care unit. Unlike the negative case reported in this study, this initiative has been sustained over time and has subsequently evolved into a broader digital platform that remains in ongoing operation. Across participant accounts, a central theme was the perceived success of co-production and sustained institutional recognition of nursing expertise. In particular, the nurse manager emphasized that the most salient experience throughout implementation was the establishment of a collaborative, “co-winning” process between clinical leadership and frontline nursing staff, alongside a consistent respect for nursing values and professional judgment. This adaptive case therefore represents a contrasting trajectory in which long-term continuity and system expansion were enabled not only by technological deployment, but by the stabilization of relational trust, professional legitimacy, and shared ownership of innovation outcomes.

| Adaptive efforts | Self-Wearing Experience and Emerging Assumptions |
| --- | --- |
| Time period | Week 2 of pilot implementation |
| Observation | Researchers and nursing managers wore wearable devices continuously for one week. Initial expectations focused on monitoring accuracy and technical performance. However, several practical challenges emerged, including skin irritation, charging inconvenience, uncertainty regarding indicator interpretation, and occasional discomfort during sleep. |
| Reflection | These experiences suggest that device acceptance may be shaped less by technical sophistication and more by comfort, convenience, and users’ understanding of device functions. |
| Analytic direction (for subsequent data collection) | How patients interpret wearable-generated physiological data  Whether physical comfort influences long-term adherence  How users evaluate perceived benefit–inconvenience trade-offs |
| Memo | Anticipating user difficulties, embodied frictions and the limits of technical determinism |

| Adaptive efforts | Bedside Data Review and Nurse Engagement |
| --- | --- |
| Time period | Week 7 of Implementation |
| Observation | Morning handovers where nursing managers shared wearable-derived sleep reports with frontline nurses.  Nurses demonstrated greater interest when sleep data revealed information unavailable through conventional monitoring systems. |
| Reflection | Engagement increased when wearable outputs were perceived as clinically actionable rather than as additional administrative burden. This suggests that perceived clinical value is a key mediator of implementation willingness. |
| Analytic Direction: | How nurses evaluate usefulness of digital health outputs  Motivational factors underlying engagement with digital technologies  Processes through which clinical relevance is constructed |
| Memo | Translating data into clinical meaning, clinical actionability as a mediator of engagement |

| Adaptive efforts | Interdisciplinary Collaboration and Sustainability |
| --- | --- |
| Time period | Week 12 of implementation |
| Observation | Ongoing collaboration among clinicians, nursing managers, and software engineers. Frequent communication occurred among clinicians, nursing managers, and software engineers. Proposed clinical modifications were often difficult for engineers to interpret, whereas technical limitations were poorly understood by clinicians |
| Reflection | Implementation challenges arise not only from technological limitations but also from structural translation gaps across disciplinary epistemologies and professional languages. |
| Analytic Direction: | Mechanisms of cross-disciplinary communication  Leadership strategies in coordinating heterogeneous expertise  Organizational integration mechanisms |
| Memo | Negotiating interdisciplinary boundaries, epistemic misalignment in interdisciplinary implementation |

**A3.2 Negative Case Analysis**

**Negative case:** high engagement but fragile sustainability in SICU wearable sleep monitoring implementation

**Overview:** An important negative case emerged from interviews describing implementation of a wearable sleep-monitoring project in a surgical intensive care unit (SICU). Although participants consistently described strong leadership support, high patient acceptance, and smooth interdisciplinary collaboration during the early implementation phase, the project ultimately failed to sustain beyond six months. Analysis of this case significantly refined the emerging grounded theory by revealing tensions between initial engagement and long-term implementation sustainability.

**Analysis:**

Participants initially described the project as highly coordinated and organizationally supported. Nursing managers, physicians, and engineers jointly participated in implementation planning, while leadership endorsement facilitated staff cooperation and reduced resistance. One participant explained:

*“Because the project was led by our leadership team, communication with colleagues was very smooth, and everyone was enthusiastic about the project.”*

Initial coding identified actions such as “mobilizing leadership legitimacy,” “aligning interdisciplinary collaboration,” and “facilitating implementation through organizational authority.” Through constant comparison, these codes contributed to the focused category of leadership-enabled implementation engagement.

Participants also emphasized that patient resistance was unexpectedly low. Nurses often introduced the wearable devices briefly, and patients generally accepted monitoring without hesitation. However, deeper analysis suggested that this apparent acceptance did not necessarily reflect meaningful engagement or reciprocal participation. One participant acknowledged:

*“We usually just put the wristband on the patient and did the questionnaire the next morning… feedback to patients was actually missing.”*

This negative case challenged early assumptions that high compliance necessarily indicated successful implementation. Focused coding identified processes including “limited reciprocal communication,” “one-directional participation,” and “restricted patient feedback integration.” These findings refined the emerging theory by demonstrating that procedural acceptance could coexist with limited patient-centred engagement.

The project further revealed tensions between technological enthusiasm and implementation burden. Although nurses perceived the monitoring system as providing more objective and scientifically useful sleep data compared with traditional observational approaches, participants simultaneously described increased workload associated with questionnaires, device management, and workflow coordination. One participant stated:

*“Additional workload definitely existed… we were already busy, and we still needed to distribute questionnaires and complete additional tasks.”*

Analysis generated initial codes including “absorbing invisible implementation labour,” “negotiating research-workflow tensions,” and “extending nursing responsibilities.” These were subsequently integrated into the focused category of hidden implementation workload.

Importantly, the eventual discontinuation of the project represented a major theoretical turning point in the analysis. Despite high organizational support during implementation, the project ended abruptly because of unsuccessful grant applications, personnel changes, and insufficient long-term infrastructural support. One participant reflected:

*“The project suddenly stopped because the related funding application was unsuccessful.”*

*Another participant noted:*

*“The wristbands are now stored in a cabinet and are no longer being used.”*

These accounts functioned as critical negative outcome cases that challenged early interpretations emphasizing leadership support and clinical enthusiasm as sufficient conditions for sustained implementation. Through theoretical comparison, the analysis shifted toward recognizing implementation sustainability as dependent on broader institutional continuity, funding stability, translational infrastructure, and long-term interdisciplinary integration.

Further negative case analysis revealed an additional structural weakness involving limited translational communication between clinical teams and engineers. Participants repeatedly described difficulty translating clinical needs into technical language. One participant explained:

*“Currently this kind of bridge role is very lacking… we can only communicate from the level of clinical needs.”*

Initial coding identified “communication asymmetry,” “absence of translational intermediaries,” and “disciplinary language fragmentation.” These findings contributed to the emerging category of fragile interdisciplinary translation capacity.

Theoretical coding ultimately demonstrated that nurse engagement in UDMT implementation was not a linear process of technological adoption, but rather a fragile and dynamic process shaped by interacting organizational, relational, infrastructural, and epistemic conditions. Negative cases played a central role in refining the substantive grounded theory by revealing that high short-term participation, leadership endorsement, and technological optimism did not necessarily translate into sustainable implementation. Instead, long-term normalization depended on continued institutional investment, stable interdisciplinary infrastructure, reciprocal patient engagement, and mechanisms capable of integrating technological systems into enduring clinical workflows.

**A3.3 Theoretical Integration (Memo Synthesis)**

Across adaptive memos and negative case analysis, the developing grounded theory indicates that implementation of wearable-based digital health technologies is a non-linear, socio-technical process shaped by interacting conditions: embodied user experience (comfort, usability); perceived clinical relevance (data meaning-making); interdisciplinary translation capacity; organizational leadership structures; hidden labor embedded in workflow integration; and institutional continuity and funding stability

**Core theoretical refinement:** Implementation sustainability is not a function of initial adoption success but of ongoing alignment across organizational, epistemic, and infrastructural domains.
