# Supplementary material for: Nurses’ Engagement in Digital Health Clinical Trials Using Unobtrusive Monitoring Technologies: Constructivist Grounded Theory Study
Source: J Med Internet Res. 2026 Sep 22;28:e90982. doi: 10.2196/90982 (PMC13596759; doi:10.2196/90982)
Supplement: Supplementary file 3 [file jmir-v28-e90982-s003.docx]

**Multimedia Appendix 3. The NURSE heuristic Framework**

Note: NURSE captures how nurses continuously: 1) Navigate uncertainty generated by ambiguous protocols, variable patient responses, and evolving technological demands; 2) Mobilize resources (technical, relational, and organizational) to sustain trial work; 3) Seek and distribute support across hierarchical and interprofessional boundaries; and 4) Sustaining empowerment under conditions of workload pressure, ethical tension, and technological friction

Memo:

The NURSE heuristic represents an analytic construction developed to interpret recurring patterns within Supported Translation. Rather than functioning as a linear or prescriptive model, it is used as a sensitizing framework to understand how nurses navigate uncertainty, mobilize available resources, seek diverse forms of support, and sustain engagement in the context of UDMT-based clinical trials. As one participant reflected, *“Even though we ran this trial for a year and faced many barriers and challenges, our nursing team kept working to overcome them. In the end, we hope it creates a win-win situation, and ideally, it also helps reduce our clinical workload and improve nursing quality” (C1-RN1).*

Across the data, these analytic elements were not experienced as discrete or sequential phases, but as interwoven and contextually responsive practices that nurses drew upon in varying combinations depending on situational demands.

- Navigating uncertainty was evident in nurses’ efforts to interpret unfamiliar technologies, evolving trial expectations, and shifting clinical responsibilities. Rather than resolving uncertainty, nurses worked within it, developing provisional understandings that allowed practice to continue.
- Mobilizing resources reflected how nurses actively drew on collective, organizational, and relational assets, including team collaboration, managerial input, and interdisciplinary communication, to translate protocol requirements into workable clinical actions.
- Seeking support further illustrated the relational nature of translation work, where institutional, technical, and emotional resources were continuously accessed to sustain participation and manage workload pressures.
- Sustaining empowerment emerged not as a final outcome, but as an ongoing orientation shaped by iterative learning, adaptation, and relational reinforcement within the clinical environment.

Overall, the NURSE heuristic captures how Supported Translation is enacted as a process of situated coherence-making, in which feasibility is continuously constructed rather than achieved as a final state.
