# Supplementary material for: Nurses’ Engagement in Digital Health Clinical Trials Using Unobtrusive Monitoring Technologies: Constructivist Grounded Theory Study
Source: J Med Internet Res. 2026 Sep 22;28:e90982. doi: 10.2196/90982 (PMC13596759; doi:10.2196/90982)
Supplement: Supplementary file 4 [file jmir-v28-e90982-s004.docx]

**Multimedia Appendix 4.**

**Table S1. Application of sensitizing frameworks in theoretical integration and refinement**

| **Framework** | **Core Constructs** | **Analytic Application in This Study** |
| --- | --- | --- |
| Normalization Process Theory | Coherence, cognitive participation, collective action, and reflexive monitoring | Used to explore how nurses made sense of unobtrusive digital monitoring technologies, built commitment to their use, and collectively operationalized trial activities within existing clinical structures. |
| Complex Adaptive Systems Theory | Agents, non-linearity, interactions, adaptation, and emergence | Provided a lens to interpret nurses’ engagement as a dynamic, evolving process shaped by feedback loops between individual readiness, organizational climate, and technological integration. |
| Sociotechnical Systems Theory | Joint optimization of social and technical subsystems; interdependence between human actors, technology, and organizational design | Provided a lens to interpret interaction between technological design, clinical workflows, and social relationships in trial implementation. |
| Innovation Theory | Diffusion, adoption, relative advantage, compatibility, and reinvention | Used to compare emerging categories with established models of technology adoption and diffusion in healthcare settings. |
