# Supplementary material for: Nurses’ Engagement in Digital Health Clinical Trials Using Unobtrusive Monitoring Technologies: Constructivist Grounded Theory Study
Source: J Med Internet Res. 2026 Sep 22;28:e90982. doi: 10.2196/90982 (PMC13596759; doi:10.2196/90982)
Supplement: Supplementary file 5 [file jmir-v28-e90982-s005.docx]

**Consolidated criteria for reporting qualitative studies (COREQ): 32-item checklist**

| **No** | **Item** | **Guide questions/description** | **Response** |  |  |  |
| --- | --- | --- | --- | --- | --- | --- |
| **Domain 1: Research team and reflexivity** | | | |  |  |  |
| Personal Characteristics | | | |  |  |  |
| 1. | Interviewer/  facilitator | Which author/s conducted the interview or focus group? | All focus groups and interviews were conducted by XW. |  |  |  |
| 2. | Credentials | What were the researcher's credentials? *E.g. PhD, MD* | Reported. XW holds a PhD in Nursing; ZZ is a PhD-trained cardiologist; LX is a senior cardiac nurse specialist. |  |  |  |
| 3. | Occupation | What was their occupation at the time of the study? | Reported. XW was a nurse researcher and Associate Professor of Nursing. |  |  |  |
| 4. | Gender | Was the researcher male or female? | Reported. XW and LX were female; ZZ was male. |  |  |  |
| 5. | Experience and training | What experience or training did the researcher have? | Reported. XW had expertise in clinical trials, digital health, and qualitative research. The research team had prior experience in qualitative methods and digital health research. |  |  |  |
| Relationship with participants | | | |  |  |  |
| 6. | Relationship established | Was a relationship established prior to study commencement? | Reported. No prior supervisory, managerial, or personal relationships existed between XW and participants. |  |  |  |
| 7. | Participant knowledge of the interviewer | What did the participants know about the researcher? e*.g. personal goals, reasons for doing the research* | Reported. Participants were informed about the researchers’ professional backgrounds, study purpose, and voluntary nature of participation. |  |  |  |
| 8. | Interviewer characteristics | What characteristics were reported about the interviewer/facilitator? e.g. *Bias, assumptions, reasons and interests in the research topic* | Reported. Reflexivity regarding professional backgrounds, assumptions, and experiences in digital health and clinical research was maintained throughout the study. |  |  |  |
| **Domain 2: study design** | | | |  |  |  |
| Theoretical framework | | | |  |  |  |
| 9. | Methodological orientation and Theory | What methodological orientation was stated to underpin the study? *e.g. grounded theory, discourse analysis, ethnography, phenomenology, content analysis* | Reported. Constructivist Grounded Theory (CGT) informed by Charmaz, with selected. |  |  |  |
| Participant selection | | | |  |  |  |
| 10. | Sampling | How were participants selected? *e.g. purposive, convenience, consecutive, snowball* | Reported. Initial purposive sampling followed by theoretical sampling and negative case sampling. Snowball sampling was used within clinical contexts. |  |  |  |
| 11. | Method of approach | How were participants approached? e*.g. face-to-face, telephone, mail, email* | Reported. Participants were invited via WeChat through nursing research departments and clinical trial teams. |  |  |  |
| 12. | Sample size | How many participants were in the study? | Reported. N = 21 nurses participated in two focus groups and individual interviews. |  |  |  |
| 13. | Non-participation | How many people refused to participate or dropped out? Reasons? | Reported. Three eligible nurses declined participation because of competing commitments or lack of interest. |  |  |  |
| Setting | | | |  |  |  |
| 14. | Setting of data collection | Where was the data collected? e*.g. home, clinic, workplace* | Reported. Private meeting rooms in participating hospitals and secure online platforms. |  |  |  |
| 15. | Presence of non-participants | Was anyone else present besides the participants and researchers? | Reported. Only participants and the researcher were present during data collection. |  |  |  |
| 16. | Description of sample | What are the important characteristics of the sample? *e.g. demographic data, date* | Reported. Participants were registered nurses directly involved in UDMT-enabled clinical trials. Sample characteristics are provided in Table 1. |  |  |  |
| Data collection | | | |  |  |  |
| 17. | Interview guide | Were questions, prompts, guides provided by the authors? Was it pilot tested? | Reported. Semi-structured interview guides were iteratively refined through ongoing analysis and theoretical sampling. |  |  |  |
| 18. | Repeat interviews | Were repeat interviews carried out? If yes, how many? | Reported. No repeat interviews were conducted. |  |  |  |
| 19  . | Audio/visual recording | Did the research use audio or visual recording to collect the data? | Reported. All focus groups and interviews were audio-recorded with consent. |  |  |  |
| 20. | Field notes | Were field notes made during and/or after the interview or focus group? | Reported. Field notes documented contextual observations, non-verbal interactions, and emerging analytic insights. |  |  |  |
| 21. | Duration | What was the duration of the interviews or focus group? | Reported. Interview duration ranged from approximately 30-65 minutes, while focus groups lasted approximately 57 minutes |  |  |  |
| 22. | Data saturation | Was data saturation discussed? | Reported. Theoretical sufficiency was achieved through ongoing theoretical sampling and negative case analysis. |  |  |  |
| 23. | Transcripts returned | Were transcripts returned to participants for comment and/or correction? | Reported. Transcripts were returned for correction. Selected participants reviewed preliminary interpretations. |  |  |  |
| **Domain 3: analysis and findings** | | | |  |  |  |
| Data analysis | | | |  |  |  |
| 24. | Number of data coders | How many data coders coded the data? | Reported. XW conducted primary coding, with LX independently coding selected transcripts. |  |  |  |
| 25. | Description of the coding tree | Did authors provide a description of the coding tree? | Reported. The coding process progressed through initial, focused, and theoretical coding. Category development is described in the Methods and appendices. |  |  |  |
| 26. | Derivation of themes | Were themes identified in advance or derived from the data? | Reported. Categories and theoretical concepts were inductively derived from the data. |  |  |  |
| 27. | Software | What software, if applicable, was used to manage the data? | Reported. Microsoft Word and Excel were used for data management and coding. |  |  |  |
| 28. | Participant checking | Did participants provide feedback on the findings? | Reported. Selected participants reviewed preliminary interpretations and theoretical explanations. |  |  |  |
| Reporting | | | |  |  | Reported. ML is a Master's student; XYW holds a PhD.  Exact Section Location: Supplementary B (Paragraph 1) |
| 29. | Quotations presented | Were participant quotations presented to illustrate the themes / findings? Was each quotation identified? e*.g. participant number* | Reported. Quotations are presented throughout the findings and identified using participant codes. |  |  |  |
| 30. | Data and findings consistent | Was there consistency between the data presented and the findings? | Reported. Findings were grounded in participants’ accounts and supported by illustrative quotations. |  |  |  |
| 31. | Clarity of major themes | Were major themes clearly presented in the findings? | Reported. Major categories and the substantive theory are clearly described. |  |  |  |
| 32. | Clarity of minor themes | Is there a description of diverse cases or discussion of minor themes? | Reported. Adaptive and disconfirming cases were included to refine category boundaries and theoretical explanations. |  |  |  |

|  | **Standards for Reporting Qualitative Research (SRQR)*** |  |
| --- | --- | --- |
|  | <http://www.equator-network.org/reporting-guidelines/srqr/> |  |
|  |  | **Page/line no(s).** |
| **Title and abstract** | |  |
|  | **Title** - Concise description of the nature and topic of the study Identifying the study as qualitative or indicating the approach (e.g., ethnography, grounded theory) or data collection methods (e.g., interview, focus group) is recommended | P3 |
|  | **Abstract** - Summary of key elements of the study using the abstract format of the intended publication; typically includes background, purpose, methods, results, and conclusions | P3-4 |
|  |  |  |
| **Introduction** | |  |
|  | **Problem formulation** - Description and significance of the problem/phenomenon studied; review of relevant theory and empirical work; problem statement | P5-7 |
|  | **Purpose or research questio**n - Purpose of the study and specific objectives or questions | P7 |
|  |  |  |
| **Methods** | |  |
|  | **Qualitative approach and research paradigm** - Qualitative approach (e.g., ethnography, grounded theory, case study, phenomenology, narrative research) and guiding theory if appropriate; identifying the research paradigm (e.g., postpositivist, constructivist/ interpretivist) is also recommended; rationale** | P7 |
|  | **Researcher characteristics and reflexivity** - Researchers’ characteristics that may influence the research, including personal attributes, qualifications/experience, relationship with participants, assumptions, and/or presuppositions; potential or actual interaction between researchers’ characteristics and the research questions, approach, methods, results, and/or transferability | P12 |
|  | **Context** - Setting/site and salient contextual factors; rationale** | P7 |
|  | **Sampling strategy** - How and why research participants, documents, or events were selected; criteria for deciding when no further sampling was necessary (e.g., sampling saturation); rationale** | P8-9 |
|  | **Ethical issues pertaining to human subjects** - Documentation of approval by an appropriate ethics review board and participant consent, or explanation for lack thereof; other confidentiality and data security issues | P15 |
|  | **Data collection methods** - Types of data collected; details of data collection procedures including (as appropriate) start and stop dates of data collection and analysis, iterative process, triangulation of sources/methods, and modification of procedures in response to evolving study findings; rationale** | P11-12 |
|  | **Data collection instruments and technologies** - Description of instruments (e.g., interview guides, questionnaires) and devices (e.g., audio recorders) used for data collection; if/how the instrument(s) changed over the course of the study | P11-12 |
|  | **Units of study** - Number and relevant characteristics of participants, documents, or events included in the study; level of participation (could be reported in results) | P10 and Table 1 |
|  | **Data processing** - Methods for processing data prior to and during analysis, including transcription, data entry, data management and security, verification of data integrity, data coding, and anonymization/de-identification of excerpts | P13 |
|  | **Data analysis** - Process by which inferences, themes, etc., were identified and developed, including the researchers involved in data analysis; usually references a specific paradigm or approach; rationale** | P13-15 |
|  | **Techniques to enhance trustworthiness** - Techniques to enhance trustworthiness and credibility of data analysis (e.g., member checking, audit trail, triangulation); rationale** | P13 |
|  |  |  |
| **Results/findings** | |  |
|  | **Synthesis and interpretation** - Main findings (e.g., interpretations, inferences, and themes); might include development of a theory or model, or integration with prior research or theory | P18-24 |
|  | **Links to empirical data** - Evidence (e.g., quotes, field notes, text excerpts, photographs) to substantiate analytic findings | P18-24 |
|  |  |  |
| **Discussion** | |  |
|  | **Integration with prior work, implications, transferability, and contribution(s) to the field -** Short summary of main findings; explanation of how findings and conclusions connect to, support, elaborate on, or challenge conclusions of earlier scholarship; discussion of scope of application/generalizability; identification of unique contribution(s) to scholarship in a discipline or field | P24-27 |
|  | **Limitations** - Trustworthiness and limitations of findings | P28 |
|  |  |  |
| **Other** | |  |
|  | **Conflicts of interest** - Potential sources of influence or perceived influence on study conduct and conclusions; how these were managed | P30 |
|  | **Funding** - Sources of funding and other support; role of funders in data collection, interpretation, and reporting | P2 |
|  |  |  |
|  | *The authors created the SRQR by searching the literature to identify guidelines, reporting standards, and critical appraisal criteria for qualitative research; reviewing the reference lists of retrieved sources; and contacting experts to gain feedback. The SRQR aims to improve the transparency of all aspects of qualitative research by providing clear standards for reporting qualitative research. |  |
|  |  |  |
|  | **The rationale should briefly discuss the justification for choosing that theory, approach, method, or technique rather than other options available, the assumptions and limitations implicit in those choices, and how those choices influence study conclusions and transferability. As appropriate, the rationale for several items might be discussed together. |  |
|  |  |  |
|  | **Reference:** |  |
|  | O’Brien BC, Harris IB, Beckman TJ, Reed DA, Cook DA. Standards for reporting qualitative research: a synthesis of recommendations. *Academic Medicine*. 2014;89(9):1245–1251. |  |
